# Occupational health outcomes among international migrant workers: a systematic review and meta-analysis

**DOI:** 10.1016/S2214-109X(19)30204-9

**Published:** 2019-05-20

**Authors:** Sally Hargreaves, Kieran Rustage, Laura B Nellums, Alys McAlpine, Nicola Pocock, Delan Devakumar, Robert W Aldridge, Ibrahim Abubakar, Kristina L Kristensen, Jan W Himmels, Jon S Friedland, Cathy Zimmerman

**Affiliations:** a Institute for Infection and Immunity, St George's, University of London, London, UK; b Section of Infectious Diseases and Immunity, Imperial College London, London, UK; c Gender, Violence and Health Centre, Department of Global Health and Development, London School of Hygiene & Tropical Medicine, London, UK; d Institute for Global Health, University College London, London; e Institute for Health Informatics, University College London, London; f Danish Research Centre for Migration, Ethnicity and Health, University of Copenhagen, Copenhagen, Denmark

## Abstract

**Background:**

Globally, there are more than 150 million international migrant workers—individuals who are employed outside of their country of origin—comprising the largest international migrant group. A substantial number of migrants work in hazardous and exploitative environments, where they might be at considerable risk of injury and ill health. However, little data on occupational health outcomes of migrant workers exist, with which to inform global policy making and delivery of health services.

**Methods:**

For this systematic review and meta-analysis, we searched Embase, MEDLINE, Ovid Global Health, and PsychINFO databases for primary research published between Jan 1, 2008, and Jan 24, 2018, reporting occupational health outcomes among international migrant workers (defined as individuals who are or have been employed outside their country of origin), without language or geographical restrictions. We excluded studies containing mixed cohorts of migrants and native workers in which migrant data could not be disaggregated, and studies that did not explicitly report migrant status. The main outcome was prevalence of occupational health outcomes (defined as any injury, mortality, or physical or psychiatric morbidity due to an individual's work or workplace environment) among international migrant workers. Summary estimates were calculated using random-effects models. The study protocol has been registered with PROSPERO, number CRD42018099465.

**Findings:**

Of the 1218 studies identified by our search, 36 studies were included in our systematic review, and 18 studies were included in the meta-analysis. The systematic review included occupational health outcomes for 12 168 international migrant workers employed in 13 countries and territories, mostly employed in unskilled manual labour. Migrant workers originated from 25 low-income and middle-income countries, and worked in the following sectors: agriculture; domestic, retail, and service sectors; construction and trade; and manufacturing and processing. Migrant workers had various psychiatric and physical morbidities, and workplace accidents and injuries were relatively common. In the meta-analysis, among 7260 international migrant workers, the pooled prevalence of having at least one occupational morbidity was 47% (95% CI 29–64; *I*^2^=99·70%). Among 3890 migrant workers, the prevalence of having at least one injury or accident, including falls from heights, fractures and dislocations, ocular injuries, and cuts was 22% (7–37; *I*^2^=99·35%).

**Interpretation:**

International migrant workers are at considerable risk of work-related ill health and injury, and their health needs are critically overlooked in research and policy. Governments, policy makers, and businesses must enforce and improve occupational health and safety measures, which should be accompanied by accessible, affordable, and appropriate health care and insurance coverage to meet the care needs of this important working population.

**Funding:**

Wellcome Trust.

## Introduction

Globally, there are more than 150 million international migrant workers—individuals who are employed, or have previously been employed outside their country of origin—comprising the largest international migrant group.^1^ Key migrant destinations are high-income countries in North America, northern, southern, and western Europe, and the Middle East,^1^ with millions of US dollars in remittances sent back to low-income and middle-income countries each year from migrant labour. Although international migration can provide opportunities for work and employment, it can also expose individuals to considerable hardship, with implications for health and wellbeing. Migrant workers, particularly those from low-income and middle-income countries, are often employed in low wage occupations with long working hours,^2^ and are likely to be employed in more dangerous jobs and industries than non-migrants.^3^ Migrants commonly work in exploitative and hazardous conditions, are more likely to be exposed to pesticides and chemicals and workplace abuse, and often have greater workloads than native workers.^2^, ^4^, ^5^

As a result of such occupational risk factors, previous research has shown migrant workers might be at increased risk of poor mental health outcomes,^6^ perinatal mortality, and increased injury compared with native workers, outcomes that are attributable to poor working and living conditions, inadequate labour protection measures, and limited entitlements to health care.^7^, ^8^ Similar to other migrant groups, migrant workers often face barriers to health care in the country to which they migrate, including limited or no access to health insurance and restrictions on their entitlement to statutory health care.^9^, ^10^ These issues have resulted in calls at the international level for targeted policies to address the health needs of migrant workers, and achieve progress towards universal health coverage in migrant populations globally.^11^

**Research in context**

**Evidence before this study**

Globally, there are more than 150 million international migrant workers—individuals who are employed outside of their country of origin—comprising the largest international migrant group. Prior to this study, we did a rapid review of PubMed for studies on international migrant workers. A systematic review of immigrant populations, work, and health summarising data on immigrant occupational health published between 1990 and 2005, showed that these populations had a high risk of occupational injuries and illnesses. The systematic review called for more global data on this population, their health needs, and approaches to improving the health of immigrant workers. A subsequent systematic review on self-perceived health across migrant groups showed that migrant workers had poorer self-reported health status than native populations.

**Added value of this study**

This is the first systematic review and meta-analysis on migration and occupational health summarising the burden of occupational morbidity among international migrant workers. Our systematic review included 12 168 international migrant workers employed in 13 countries and territories from 25 low-income and middle-income countries, mostly employed in unskilled manual labour. The research is a robust and comprehensive examination of the existing peer-reviewed primary evidence base, providing insight into the occupational risk factors and health outcomes of migrant workers. We found that migrant workers are at significant risk of work-related ill health and injury. Migrant workers had various physical and psychiatric morbidities, and workplace accidents and injuries were relatively common. The findings provide important new insights into the health implications of existing employment conditions and entitlement to care worldwide, and highlight the need to continue to promote global frameworks such as the WHO Global Plan of Action on Workers' Health (2008–17), and to strengthen and monitor national policies to ensure the protection and adequate care of international migrant workers.

**Implications of all the available evidence**

International migrant workers have a high burden of physical and psychiatric morbidity, including accidents and injury, as a result of employment in a foreign country, and to date, their health needs have been critically overlooked in research and policy. Although more robust, standardised, and comparable research is needed to further examine such outcomes and associated risk factors, there must now be a focus on ensuring occupational health and safety policies are in place and enforced for the benefit of international migrant workers. Governments, policy makers, and businesses must work to develop and enforce occupational health and safety measures, and promote access to health care and insurance coverage. Health services in migrant-receiving countries might need to be adapted and developed to meet the care needs of this important working population. Existing international frameworks must continue to be promoted and implemented, and further systematic and societal changes ensuring equitable access to health care for international migrant workers are also necessary to prevent adverse outcomes and protect the health of migrant workers.

Consistent with the UN's Sustainable Development Goals, there has been a renewed commitment to improve working conditions, occupational health, and universal health coverage and access to services in all populations, including migrants. Specifically, Sustainable Development Goal 8 promotes decent work and economic growth, committing to “protect labour rights and promote safe and secure working environments for all workers, including migrant workers”.^12^ This development goal also places focus on providing adequate workplace health and safety, and protection against violence and exploitation for migrant workers. The protection of migrant workers has also been highlighted in key international frameworks, including the WHO Global Plan of Action on Workers' Health (2008–17),^13^ which endorses a global strategy for occupational health for all.

To date, however, little research has been done on the occupational health risks, outcomes, and resulting health service needs in migrant workers, and little evidence exists about the persisting health needs of these populations in accordance with global frameworks. Therefore, we did a systematic review and meta-analysis of the occupational health outcomes among international migrant workers worldwide. The specific aim of the meta-analysis was to summarise the global prevalence of occupational morbidity in migrant workers, and describe occupational health risks and outcomes associated with specific industries. These findings are intended to promote global and national policy responses, across all migrant-receiving countries and for all migrant worker groups, and ensure that health services in migrant-receiving countries can be adapted and developed to meet the care needs of this important working population.

## Methods

### Search strategy and inclusion criteria

For this systematic review and meta-analysis, we searched Embase, MEDLINE, Ovid Global Health, and PsychINFO databases for primary research published between Jan 1, 2008, and Jan 24, 2018, reporting occupational health outcomes among international migrant workers without language or geographical restrictions, using a Boolean search strategy developed by consulting previous literature,^14^ and experts in the field (appendix p 8). Full search terms are provided in the appendix (p 8).

We defined occupational health outcomes as any injury, mortality, or physical or psychiatric morbidity reported as a result of an individual's work or work environment and exposure to workplace-related physical and psychosocial risks.^15^ We adapted the International Labour Organization definition of migrant workers,^16^ to include individuals employed outside their country of origin, regardless of legal or immigration status. We included these individuals to ensure that our systematic review would include data on diverse populations with various reasons for migration, and undocumented migrant workers, who are likely to have fewer legal protections, and be at increased risk of exploitation.

We searched for peer-reviewed primary research published after the publication of the WHO Global Plan of Action on Workers' Health (2008–17) to capture the persisting occupational health outcomes occurring in this group, and to inform future strategies for policy and practice to address remaining disparities. Studies published after 2008 but reporting data obtained before 2008 were excluded, unless these findings were disaggregated by year of data collection. Studies containing mixed cohorts of international migrants and native workers were excluded unless occupational health outcomes were disaggregated by migrant status. We also excluded studies that did not explicitly report migrant (ie, foreign-born) status—eg, studies that used ethnicity as a proxy for migrant status. We also excluded papers that solely examined the impact of employer-provided housing, or the impact of infectious diseases such as malaria and tuberculosis, since these conditions are not necessarily attributable to occupational exposures.

Four reviewers (KR, NP, AMc, KLK) screened the titles and abstracts. Each text was initially independently screened by two reviewers (KR and KLK) using the web-based application, Rayyan,^17^ with disagreements resolved by a third reviewer (SH).

This review was done in accordance with the Preferred Reporting Items for Systematic Review and Meta-Analysis (PRISMA) guidelines.^18^

### Data analysis

Two independent reviewers (KR and KLK) screened full texts and extracted data, with discrepancies and disagreements resolved by discussion. We extracted data on publication date, migrant worker population, participant socio-demographic characteristics (where available), employment sector, country of origin, employment or study country, occupational health outcomes, and study type using data extraction forms. When multiple publications were identified that reported on the same populations and outcomes, only the most comprehensive study was included in the meta-analysis to avoid duplication of data.

We used the Metaprop command in the statistical software Stata (version 13) to calculate the pooled prevalence of morbidity (including any reported occupational health outcome), and accident and injury and corresponding 95% CIs, specifically among international migrant workers.^19^ Heterogeneity between studies was assessed using the *I*^2^ statistic. We predicted high levels of heterogeneity, and therefore did summary estimates with random-effects models. We assessed the heterogeneity in key characteristics including date of publication, region of origin, destination, employment sector, health outcome, and study quality.

Each study was also categorised by the industry or employment sector of the included populations. We defined four groups of international migrant workers, on the basis of professions included in the retrieved studies: agricultural labour; construction and trade; domestic, retail, and service sector; and manufacturing and processing. Using these constructs, we provide a descriptive analysis of common occupational health outcomes among migrants in these professions, and factors identified to be associated with such outcomes. Meta-analyses were also done by industry if data were available. The meta-analyses were done to provide pooled estimates of morbidity and injury across diverse sectors, and visualisations of the heterogeneity in the evidence base and the diversity of outcomes and migrant workers represented.

Study quality was assessed using the Joanna Briggs Institute Checklist for Prevalence Studies,^20^ which enabled assessment of included studies in relation to risk of bias, rigour, and transparency. Studies scoring 1–3 were defined as low quality, 4–6 as average quality, and 7–9 as high quality. Studies were not excluded on the basis of their quality score to increase transparency and to ensure all available evidence in this area was reported. However, we did sensitivity analyses to examine the effect of study quality on the meta-analysis, whereby low and average quality studies were excluded.

This study is registered with PROSPERO, number CRD42018099465.

### Role of the funding source

The funders of the study had no role in study design, data collection, data analysis, data interpretation, writing of the report, or the decision to submit the paper for publication. All authors had full access to all data in this study and had responsibility for the decision to submit for publication.

## Results

**Figure 1:**
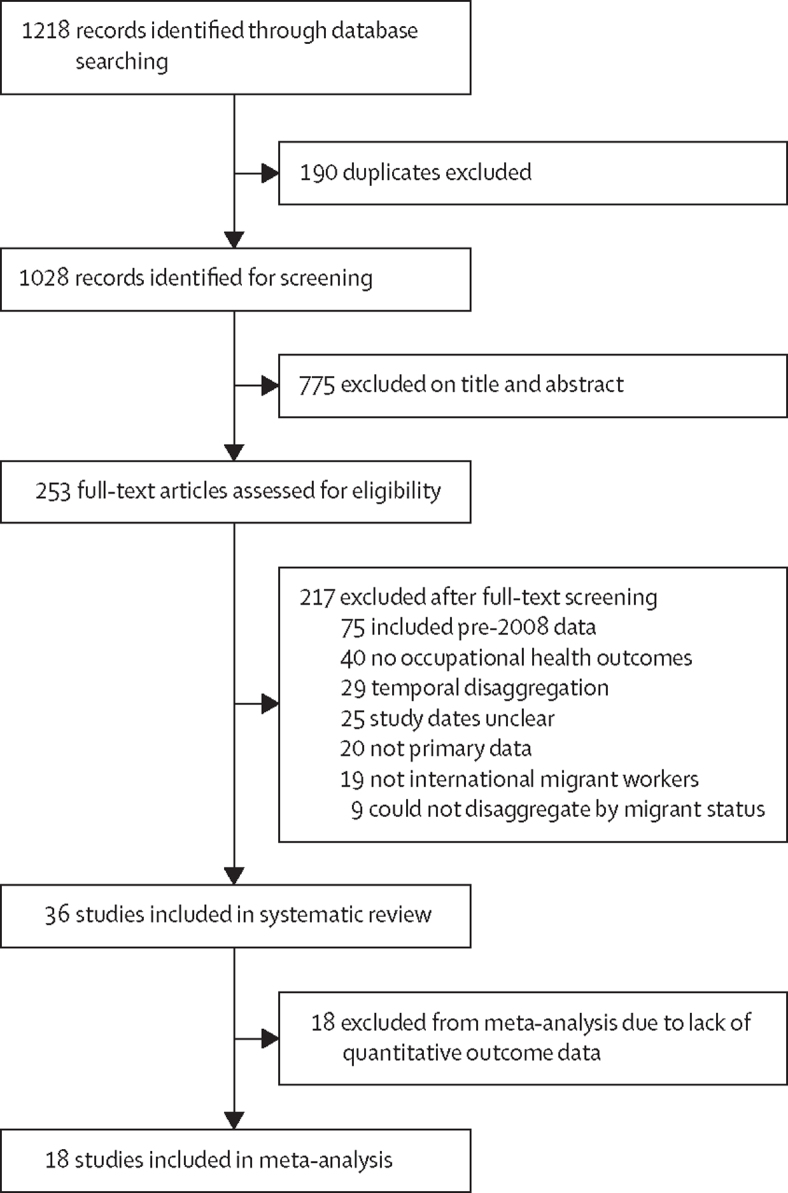
Study selection

We identified 1218 publications, of which 190 were duplicates. 1028 articles were screened for eligibility, of which 253 were included in the full-text screening (figure 1). 36 studies^21^, ^22^, ^23^, ^24^, ^25^, ^26^, ^27^, ^28^, ^29^, ^30^, ^31^, ^32^, ^33^, ^34^, ^35^, ^36^, ^37^, ^38^, ^39^, ^40^, ^41^, ^42^, ^43^, ^44^, ^45^, ^46^, ^47^, ^48^, ^49^, ^50^, ^51^, ^52^, ^53^, ^54^, ^55^, ^56^ met our inclusion criteria for the systematic review (table) and 18 articles^21^, ^22^, ^26^, ^27^, ^28^, ^29^, ^31^, ^33^, ^38^, ^41^, ^43^, ^46^, ^47^, ^48^, ^49^, ^51^, ^52^, ^54^ were included in the meta-analyses.

**Table:** Characteristics of studies included in the systematic review of occupational health outcomes among international migrant workers

	**Study year**	**Location**	**Study design**	**Industry or profession**	**Health focus**	Quality score*
Agudelo-Suárez et al^51^	2009	Spain	Cross-sectional	Unknown	Mixed outcomes	7
Al-Maskari et al^50^	2011	United Arab Emirates	Cross-sectional	Construction and trade; domestic, retail, and services	Depression and suicidal behaviour	8
Anjara et al^39^	2017	Singapore	Cross-sectional	Domestic, retail, and services	Stress, health, and quality of life	7
Arcury et al^32^	2016	USA	Cross-sectional	Agricultural	Mixed outcomes	6
Arcury et al^33^	2012	USA	Cross-sectional	Agricultural	Musculoskeletal discomfort, working while injured, and depression	6
Arici et al^44^	2016	Italy	Cross-sectional	Domestic, retail, and services; manufacturing and processing	Health inequalities due to work-related psychosocial risk factors	3
Baker and Chappelle^56^	2012	USA	Cross-sectional	Agricultural	Mixed outcomes	7
Bener^47^	2017	Qatar	Cross-sectional	Agricultural; construction and trade; domestic, retail and services	Self-reported general health	6
Brumitt et al^21^	2010	USA	Cross-sectional	Agricultural	Musculoskeletal pain	5
Capasso et al^45^	2016	Italy	Cross-sectional	Construction and trade; domestic, retail, and services; manufacturing and processing	Stress and subjective self-perceived health	8
Cartwright et al^23^	2014	USA	Cross-sectional	Agricultural	Carpal tunnel syndrome	7
Palupi et al^55^	2017	Taiwan	Cross-sectional	Domestic, retail, and services	Depressive symptoms, fatigue	8
Fernández-Esquer et al^24^	2015	USA	Cross-sectional	Construction and trade; domestic, retail, and services	Self-reported injuries	7
Flunker et al^26^	2017	USA	Cross-sectional	Agricultural	Pulmonary function and respiratory symptoms	8
Gao et al^52^	2014	Hong Kong	Cross-sectional	Domestic, retail, and services	Oral or dental health	7
Harrigan et al^40^	2017	Singapore	Cross-sectional	Unknown	Mental health	6
Joshi et al^46^	2011	Qatar, Saudi Arabia, United Arab Emirates	Cross-sectional	Agricultural; construction and trade; domestic, retail, and services	General or mixed health outcomes	6
Korkmaz and Park^49^	2018	South Korea	Cross-sectional	Construction and trade	Workplace injury or accidents	7
Lee et al^48^	2011	South Korea	Cross-sectional	Unknown	Psychosocial factors and musculoskeletal disorders	7
Lee et al^38^	2014	Singapore	Cross-sectional	Construction and trade	General or mixed health outcomes	8
Pichardo-Geisinger et al^34^	2014	USA	Cross-sectional	Agricultural	Tinea pedis and onychomycosis infections	7
Pichardo-Geisinger et al^35^	2013	USA	Cross-sectional	Agricultural	Dermatological conditions	8
Quach et al^27^	2013	USA	Cross-sectional	Domestic, retail, and services	Nose, throat, and skin irritation, headaches and coughs associated with chemical exposure	6
Quandt et al^22^	2012	USA	Cross-sectional	Agricultural	Eye and ocular injuries	5
Quandt et al^30^	2013	USA	Cross-sectional	Agricultural	Mixed health outcomes	5
Quandt et al^31^	2014	USA	Cross-sectional	Agricultural	Dermatological conditions and associated quality of life	7
Rathod^28^	2016	USA	Cross-sectional	Construction and trade; domestic, retail, and services.	Mixed health outcomes	8
Riley et al^25^	2016	USA	Cross-sectional	Domestic, retail, and services	Sleep frequency, duration, and quality	6
Sandberg et al^29^	2012	USA	Cross-sectional	Agricultural	Excessive daytime sleepiness, depression, and musculoskeletal pain	7
Santos et al^54^	2014	Malaysia	Cross-sectional	Manufacturing and processing	Musculoskeletal pain	7
Schulz et al^36^	2013	USA	Cross-sectional	Agricultural	Musculoskeletal pain	8
Thetkathuek et al^42^	2017	Thailand	Cross-sectional	Agricultural	Musculoskeletal pain	7
Soe et al^41^	2015	Thailand	Cross-sectional	Manufacturing and processing	Musculoskeletal pain	6
Tomita et al^43^	2010	Thailand	Cross-sectional	Manufacturing and processing	Musculoskeletal pain	8
Winkelman et al^37^	2013	USA	Cross-sectional	Agricultural	Stress and depression	5
Zahreddine et al^53^	2013	Lebanon	Cross-sectional	Domestic, retail, and services	Psychiatric morbidity	7

* Study quality was assessed using a nine point scale, whereby studies scoring 1–3 were defined low quality, 4–6 as average quality, and 7–9 as high quality.

All 36 studies included in the systematic review had a cross-sectional design, used a combination of qualitative and social research methodologies, such as interviews and surveys, and included data for 12 168 international migrants employed in 13 countries and territories: USA (n=17),^21^, ^22^, ^23^, ^24^, ^25^, ^26^, ^27^, ^28^, ^29^, ^30^, ^31^, ^32^, ^33^, ^34^, ^35^, ^36^, ^37^ Singapore (n=3),^38^, ^39^, ^40^ Thailand (n=3),^41^, ^42^, ^43^ Italy (n=2),^44^, ^45^ Qatar (n=2),^46^, ^47^ South Korea (n=2),^48^, ^49^ United Arab Emirates (n=2),^46^, ^50^ Spain (n=1),^51^ Saudi Arabia (n=1),^46^ Hong Kong (n=1),^52^ Lebanon (n=1),^53^ Malaysia (n=1),^54^ and Taiwan (n=1).^55^ Although country of origin and migrant number from different origin countries was not always reported, international migrants in the included studies originated from 25 countries: Ecuador,^51^ Morocco,^45^, ^51^ Romania,^51^ Colombia,^51^ Thailand,^48^ Vietnam,^27^, ^48^, ^49^ Philippines,^25^, ^39^, ^47^, ^48^, ^53^ India,^38^, ^47^, ^50^, ^54^ Bangladesh,^38^, ^47^, ^50^, ^53^ Pakistan,^47^, ^50^ Nepal,^46^, ^47^, ^53^, ^54^ Mexico,^34^, ^37^, ^56^ Guatamala,^28^, ^34^, ^37^, ^56^ Indonesia,^39^, ^52^, ^54^, ^55^ Ethiopia,^53^ Tonga,^53^ Sri Lanka,^47^, ^53^, ^54^ Myanmar,^43^, ^54^ North Korea,^47^ Cambodia,^42^ China,^49^ Kazakhstan,^49^ El Salvador,^28^ Honduras,^28^ and Ghana.^45^ Several studies^21^, ^23^, ^24^, ^26^, ^29^, ^31^, ^32^, ^33^, ^34^ did not report the nationality of included individuals, instead including them in broader categories, such as Latino or Hispanic (table).

The identified studies predominantly described occupational health outcomes among migrant workers employed in unskilled manual labour. We categorised the professions of included migrants into four groups: 16 studies^21^, ^22^, ^23^, ^26^, ^29^, ^30^, ^32^, ^33^, ^34^, ^35^, ^36^, ^37^, ^42^, ^46^, ^47^, ^56^ included agricultural workers; 13 studies^24^, ^25^, ^27^, ^28^, ^39^, ^44^, ^45^, ^46^, ^47^, ^50^, ^52^, ^53^, ^55^ included domestic, retail, and service sector workers; eight studies^24^, ^28^, ^38^, ^45^, ^46^, ^47^, ^49^, ^50^ included construction and trade labourers; and five studies^41^, ^43^, ^44^, ^45^, ^54^ included manufacturing and processing workers.

Research on migrants employed in agriculture most commonly examined musculoskeletal pain,^21^, ^29^, ^33^, ^36^, ^42^, ^56^ dermatological conditions,^31^, ^34^, ^35^ and depression.^29^, ^33^, ^37^, ^56^ Health problems were associated with employment in the agriculture or construction sectors, whereby individuals in these professions were more likely to have an accident or injury at work than those employed in other professions.^46^ In studies of agricultural workers, musculoskeletal pain or injury was common, with prevalence estimates ranging from 5% (15 of 300 migrant workers)^29^ to 48·4% (139 of 287 migrant workers).^21^ These estimates highlight the diversity across studies, both within these two studies examining similar outcomes in the same occupational sector,^21^, ^29^ and other studies of agricultural workers assessing distinct outcomes such as pulmonary function and respiratory symptoms, which Flunker and colleagues^26^ reported in 79% of workers. In some studies, prevalence of musculoskeletal injury was associated with older age,^21^ working more than 40 h per week,^36^ working in agriculture for less than 10 years,^42^ working posture,^42^ and a poor work safety climate.^33^

Dermatological infections were also common.^31^ Among 518 migrant workers in North Carolina (USA), the prevalence of onychomycosis infection was 32·0% and the prevalence of tinea pedis fungal infection was 37·8%, and infectious skin diseases were reported among 55·8% of workers.^34^, ^35^ Dermatological conditions were attributable to the occlusive footwear worn by agricultural workers,^34^ and associated with younger age, male sex, and working as a poultry processor.^35^

Symptoms of depression were prevalent among migrant agricultural workers.^56^ Almost a third (82 [285] of 294) of migrant farmworkers in North Carolina had reported depressive symptoms (Center for Epidemiologic Studies Depression Scale score ≥10), which was positively associated with increased day time sleepiness, and number of years employed in agriculture.^29^

Studies including domestic workers, retail, and services sector employees reported outcomes associated with depression,^50^, ^55^ stress,^39^, ^45^ and other psychiatric problems,^44^, ^53^ in addition to physical outcomes.^25^, ^28^, ^46^, ^52^ Across these studies, the prevalence and type of reported outcomes varied considerably, ranging from 19% of workers in Qatar with a self-reported health problem,^47^ to 94% of workers in Hong Kong reporting oral or dental health needs.^52^

Among retail and service sector employees, mental health problems were common. Riley and colleagues^25^ reported the detrimental effect of domestic live-in care work on the quality and time of sleep among Filipino live-in carers in Los Angeles (USA). Female migrant domestic workers in Singapore reported a high prevalence of symptoms of stress (53%), and social isolation (20%), with stress associated with worsening quality of life and isolation. Despite these findings, 146 (80%) of 180 of the women reported being satisfied with their health.^39^ Palupi and colleagues^55^ reported that among 194 Indonesian women working domestically in Taiwan, 54 (28%) reported fatigue symptoms and 72 (37%) reported depressive symptoms. Symptoms of depression were associated with fatigue and poor working conditions. In a study of 33 female migrant domestic workers who had been admitted to hospital in Lebanon, high levels of abuse were reported, with 50·0% having experienced verbal abuse (including racist insults), 37·5% reporting physical abuse, and 12·5% reporting sexual assault. Most abuse was reportedly perpetrated by employers.^53^

Health outcomes among labourers in construction and trades included physical^24^, ^38^, ^45^, ^46^, ^47^ and psychiatric morbidity.^45^, ^50^

Within the studies of migrant workers exclusively employed in construction and trades, body aches, joint pains, and injuries were common. For example, workplace injuries and accidents were reported by 32 (6·15%) of 525 migrant workers in a cohort in Singapore,^38^ and 13 (9·3%) of 140 workers in South Korea.^49^ However, the South Korean study reported no significant difference in injury prevalence between migrant and non-migrant worker populations, but factors such as age, education, and training were associated with accident rates across all individuals.^49^

Low wages, long working hours, construction work, and physical illness were also found to be associated with depression in a study of individuals from different sectors, including construction workers, mechanics, and carpenters.^50^ An increased prevalence of interpersonal disorders (defined as interpersonal sensitivity, paranoid ideation, obsessive-compulsive behaviour, and hostility) and other anxious depressive disorders^45^ were associated with employment in the construction industry. In another study of Nepalese migrants employed in Qatar, Saudi Arabia, and the United Arab Emirates, 231 (56·6%) of 408 migrant workers reported a health problem in the previous 12 months of employment. Health problems were more prevalent among construction workers, who were more likely to have an accident or workplace injury compared with other professions.^46^

Five studies included outcomes on international migrant workers working in manufacturing and processing industries: three^41^, ^43^, ^54^ included international migrant workers employed exclusively within these industries, and two^44^, ^45^ included individuals working in mixed industries. The health outcomes explored in these studies included musculoskeletal pain,^41^, ^43^, ^54^ work-related stress, and self-reported health and wellbeing.^45^

Among the studies reporting on musculoskeletal pain, a range of symptoms were reported. In two studies done in Thailand, 13 (16·3%) of 80^43^ and 108 (29·4%) of 368^41^ migrant seafood processing factory workers reported lower back pain during the past week. The prevalence of musculoskeletal pain was also high among manufacturing employees (166 [45·1%] of 368).^41^ In a study of migrant workers in the Malaysian manufacturing industry,^54^ nearly two thirds of workers (204 [64·4%] of 317) reported that musculoskeletal pain was a problem. Previous studies suggest that the following factors are significantly associated with musculoskeletal pain: older age (>40 years);^43^ a heavy workload;^54^ unsociable or long hours;^41^, ^54^ history of injury;^43^ poor health;^41^, ^43^ problematic and unreliable machinery;^54^ marital status and the number of dependents of an individual;^43^ and having an awkward posture and prolonged periods of standing during work.^41^, ^43^

Studies^44^, ^45^ that included manufacturing and processing workers as part of a mixed cohort of professions showed that migrants had a higher prevalence of lower back and upper limb musculoskeletal disorders, and a higher prevalence of interpersonal disorders than non-migrant populations.

**Figure 2:**
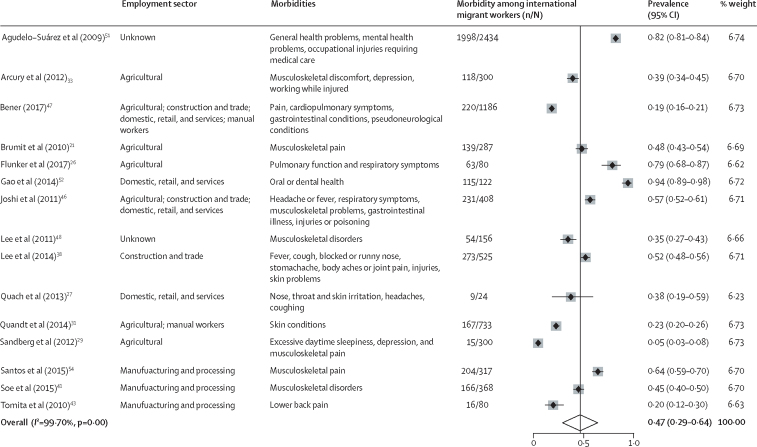
Forest plot of prevalence of having at least one reported occupational health outcome among international migrant workers

**Figure 3:**
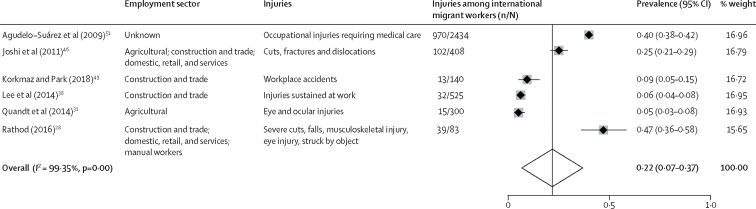
Forest plot of prevalence of having at least one occupational accident or injury among international migrant workers

Data for 7260 and 3890 migrant workers were available for the meta-analyses of morbidity and injury, respectively. Overall, the pooled prevalence of having at least one reported occupational morbidity among 7260 migrant workers was 47% (95% CI 29–64; *I*^2^=99·70%; figure 2). Among 3890 migrant workers, the estimated pooled prevalence of having at least one reported injury or accident was 22% (95% CI 7–37; *I*^2^=99·35%; figure 3). Meta-analyses were also done to examine the prevalence of occupational morbidity by industry (appendix pp 2–8). There were no clear patterns found in rates of morbidity or injury associated with factors such as study date, region of origin, sector, or destination country. Considerable variation in the types and prevalence of outcomes was observed across occupational sectors (figure 2, figure 3).

We did sensitivity analyses to examine the effect of article quality score on estimated prevalence of occupational morbidity or injury across included articles. When we excluded low and average quality studies, the pooled prevalence of occupational morbidity (50%, 95% CI 25–76; *I*^2^=99·78%) and injury (21%, 4–39; *I*^2^=99·47%) did not differ significantly from the estimated prevalence when all studies were included (appendix pp 6, 7).

The exact number and type of accidents or injuries were not always clear, because publications most commonly categorised injury or accident as one outcome. However, where described, accidents and injuries included ocular injuries,^22^, ^28^ cuts,^28^, ^46^ falls from height,^28^ and fractures and disclocations.^46^ In one study including 2434 migrant workers,^51^ 23% had reported an occupational injury that required medical attention. These data suggest that international migrant workers are often employed in sectors with a high risk of injuries and accidents and that they have various health needs associated with their occupations. Although high rates of occupational morbidity and injury were reported, little data on mortality among migrant workers was available.

## Discussion

This systematic review and meta-analysis shows high rates of morbidity, injury, and accidents worldwide among international migrant workers. This study is predominantly representative of migrant workers employed in manual labour occupations, with low wages and long working hours, and undocumented migrants. The findings highlight that migrant workers in such occupations, who might include a diverse group of individuals ranging from forced migrants to economic migrants, continue to be at risk of work-related ill health and injury, even in the context of international frameworks such as the WHO Global Plan of Action on Workers' Health. Migrants had a range of physical and psychiatric morbidities, and workplace injuries and accidents were relatively common. In the meta-analyses, 47% of international migrant workers had occupational morbidities, and 22% of migrant workers had reported a workplace injury or accident. The findings of included studies were heterogeneous, with some research showing no difference in occupational health outcomes such as injury between migrant and native workers,^49^ whereas other studies^44^, ^45^ showed migrant workers to be at increased risk of physical and psychiatric morbidity compared with non-migrant labourers. However, our systematic review and meta-analysis highlights that international migrant workers continue to be at considerable risk of harm and ill-health as a consequence of their workplace environment.

Our findings are consistent with a previous review^14^ investigating occupational outcomes among migrant workers, which highlighted that these individuals are generally at high risk of occupational injuries and illnesses. Similar to our research, when examining risk between migrant and native workers, the review identified inconsistent findings. Although some included studies reported similar rates of injury in migrant and native workers, others highlighted differences with regard to injury type, or key risk factors such as gender, time since arrival in destination country, or specific industry. The review also suggested that mortality rates from injuries might be higher among migrant workers than non-migrant workers.^14^ A systematic review^57^ examining self-perceived health across a range of migrant populations identified that migrant workers had poorer self-reported health than native populations, which is consistent with some of the findings in this study.

Poor self-reported health outcomes among migrants might be associated with factors less proximally linked to immediate occupational risk exposures, such as limited employment rights, restricted access to health care and social welfare in destination countries,^9^, ^10^ and difficulties in acculturation and adapting to host countries.^58^, ^59^ Furthermore, migrant workers might be at greater risk of other key social determinants such as poor housing or living conditions, which might contribute to their risk of other health outcomes. Certain occupational sectors could also be more representative of specific migrant groups (eg, employment of one sex or migrants from specific geographical regions), which might also be predictors of occupational risk or specific outcomes.^45^, ^60^ Across the included studies, few data were available on secondary health outcomes, or migrant characteristics, limiting our ability to formally explore these factors in the meta-analyses, although such factors have been highlighted in the descriptive analysis of the included studies.

Previous research also indicates an association between factors such as harassment, exploitation, violence, or discrimination and poor health outcomes, particularly mental health outcomes.^61^, ^62^ Integration within destination countries has also been shown to be an important predictor of migrant health, which might include both integration into health services and the labour market, and wider social integration.^63^, ^64^ Data examining the association between these factors and occupational health outcomes were scarce. Additionally, little information was available about how frequently occupational health needs were reported by migrants, how well they were recorded, and whether compensation was provided to migrants with occupational morbidities. Further research into these factors is needed to inform strategies to improve employment conditions and occupational risk in migrant workers.

Overall, as highlighted in our study, few studies include data on health outcomes for both migrant and native workers, since most of the identified studies only included data on migrant populations. As a result, comparisons between migrants and non-migrants are limited in existing global data. In our systematic review and meta-analysis, little mortality data were available for migrant workers. This paucity of data is likely to be partly attributable to reporting bias in the peer-reviewed primary data in this review, with mortality data typically being reported in national registry data, and thus not captured accurately. Additionally, mortality is likely to be under-reported in migrant populations, since non-residents and transient populations might not be included in national mortality statistics. The level of under-reporting might be even higher for more marginalised migrant groups, such as forced or undocumented migrants. Individuals have also been hypothesised to return to countries of origin as a result of illness or old age (referred to as the salmon bias), although evidence is inconsistent. Overall, the scarcity of mortality data for migrant workers represents an important gap in the literature. These factors might also contribute to under-reporting of morbidity in these populations, and thus poor occupational health outcomes might also be under-reported. This might be exacerbated by recruitment and selection bias, with individuals who are more marginalised (eg, due to being a forced or undocumented migrant) or who face greater barriers to engagement in research (eg, language, legal status, health status, fear) less likely to be represented.

The high prevalence of morbidities, in addition to injuries and accidents reported by migrant workers, reinforces the fact that greater progress toward universal health coverage (as outlined by the Sustainable Development Goals)^65^ and worker rights are still urgently needed. A 2017 WHO report^11^ on female migrant domestic workers highlights the precarious legal situation and limited rights many workers have in destination countries (including low wages, long hours, and a lack of health insurance), which ultimately impact the ability of individuals to access health care. Such experiences were mirrored frequently within the studies included in this systematic review and meta-analysis. Hostile employers,^28^ abuse and assault by employers,^53^ threats of deportation,^40^ fear of deportation,^56^ racial discrimination,^45^ poor health and safety practices,^27^, ^28^ lack of health insurance,^38^, ^46^ delayed treatments,^22^ and a poor awareness of rights and entitlements overall,^47^ were evident in included studies. Such findings reinforce the narrative in which migrant workers are often exploited, which is likely to contribute to the prevalence of illness and injury found in this study. Meaningful improvements in the health of migrant workers will only be achieved when their rights and access to health care are guaranteed. Efforts should be directed towards ensuring that occupational health and safety policies are implemented and enforced in migrant-receiving countries, in line with a global commitment to reduce health disparities, promote healthy workplaces, and ensure universal health coverage.

This study provides a comprehensive summary of the burden of occupational morbidity and injury among migrant workers worldwide, and demonstrates the persisting occupational risk factors and resulting poor health outcomes of this important working population. The key strengths of this study include the robust systematic methodological approach used and meta-analysis of available data to provide the first estimates for morbidity and injury among international migrant workers in a global context.

However, the evidence base has several limitations that should be considered. The results largely represent international migrants moving from lower-income to higher-income countries: 17 of the 36 included studies were done in the USA, and three were done in Europe. This imbalance is likely to be driven by both barriers to publishing research that might exist in low-income or middle-income settings (eg, research language, funding resources to pay publication fees), and the fact that more research in this field is done in higher-income settings (due to funding resources, prioritisation of this research area, and employment laws). As a result, the findings might not accurately represent the experiences of migrants moving between high-income countries, or between low-income and middle-income countries, which constitutes the majority of migrant worker flows globally.^1^ The finding that occupational health outcomes did not differ according to destination country might be partly attributed to a gap in the evidence base around the health of migrant workers in low-income and middle-income countries. Poor occupational health outcomes might also be reported less frequently in countries with fewer resources or less robust employment regulations. Despite this, such limitations are indicative of this field of research, with a bibliometric analysis of publications in this area revealing that data from lower-income countries of origin, and from Asia, Latin America, Africa, the Middle East, and eastern Europe are scarce, despite the high levels of migration observed in these regions.^66^ Additionally, internal migrants were not included, who also represent a large migrant worker population.

Additionally, this study predominantly identified literature reporting on individuals working in manual labour or poorly paid jobs with long working hours, and in turn, focuses on specific migrant populations overrepresented within these sectors. As a result, little data were available on occupational health outcomes among migrants in high-skilled or professional sectors. Undocumented migrants were also under-represented across the included research, which is consistent with other areas of migrant health research in which these groups are under-represented as a result of barriers to participation or health-care utilisation because of their precarious legal status. Undocumented migrant workers might be at increased risk of poorer occupational outcomes, and under-reporting of these outcomes, because of their legal status, limited legal protection, and increased risk of exploitation, and thus the findings might not represent the even greater needs of this particular group.

Our findings are further limited by the heterogeneity of the included studies, partly attributed to substantial variations in methods used, occupational outcomes examined, and populations included. Since no standardised approach exists for measuring or reporting occupational health outcomes among migrant workers, our review likely represents an underestimation of the total range or extent of morbidities among international migrant workers. The quality assessment of the included studies provides insight into study quality; however, it is also important to acknowledge the limitations associated with cross-sectional data, which might have both contributed to the heterogeneity across the included studies, and also make it challenging to elucidate any association between migrant status, occupational risk factors, and health outcomes.

Although considerable limitations exist in the evidence base, this systematic review and meta-analysis provides a comprehensive examination of the available data. The meta-analysis serves both to highlight, at an aggregated level, the high burden of occupational morbidity across the diverse range of sectors and migrant worker groups represented. Furthermore, considering the substantial heterogeneity in this field, the forest plots provide a visual representation of the variation in health outcomes of migrant workers across employment sectors, and the need to harmonise the collection and reporting of data on occupational health outcomes in this field. Ultimately this work highlights the need to continue efforts to improve employment conditions and protection for all migrant workers, but also to strengthen the evidence base and establish common approaches to measuring and assessing health outcomes among these populations.

To date, the health needs of migrant workers have been overlooked in research and policy. Governments, policy makers, and businesses must improve occupational health and safety measures, and promote entitlement to statutory health care and insurance coverage. Health services in migrant-receiving countries might need to be adapted and developed to meet the care needs of this working population. Areas for future research and next steps are outlined in the panel. More robust research, using standardised methodologies and reporting, and requirements to make data freely available or open access, are now needed to accurately assess the health needs of international migrant workers and explore options for both national service delivery and policy direction. Such progress might be supported by the use of a common framework for classifying the occupations of workers (eg, the International Standard Classification of Occupations),^67^ and for the measurement of health outcomes and morbidity (in accordance with the International Classification of Diseases, 11th revision^68^), across all studies examining health outcomes in migrant workers to ensure more directly comparable results.

Ultimately, a holistic global response is necessary to ensure that adverse occupational health outcomes among migrant workers are improved. Such a response will require a robust and evidence-based approach to prevent and monitor the occupational risk factors and associated outcomes, and has been prioritised in research and policy, including the UCL-*Lancet* Commission on Migration and Health,^69^ and the Global Compact for Migration.^70^ Such efforts must also be accompanied by the introduction of new policies and enforcement of existing policies to protect migrant workers in the workplace, and ensure equitable access to health care. The findings provide important new insights into the health implications of labour migration and highlight the need to continue to promote global frameworks such as the WHO Global Plan of Action on Workers' Health (2008–17), and to strengthen and monitor national policies to ensure the protection of international migrant workers.
