# Supplementary material for: Occupational health outcomes among international migrant workers: a systematic review and meta-analysis
Source: Lancet Glob Health. 2019 May 20;7(7):e872–82. doi: 10.1016/S2214-109X(19)30204-9 (PMC6565984; doi:10.1016/S2214-109X(19)30204-9)
Supplement: Supplementary appendix [file mmc1.pdf]

THE LANCET
Global Health

## Supplementary appendix

This appendix formed part of the original submission and has been peer reviewed. We post it as supplied by the authors.

Supplement to: Hargreaves S, Rustage K, Nellums LB, et al. Occupational health outcomes among international migrant workers: a systematic review and meta-analysis. *Lancet Glob Health* 2019; published online May 20. http://dx.doi.org/10.1016/S2214-109X(19)30204-9.

**Appendix**

***Occupational health outcomes among international migrant workers: a systematic review and meta-analysis***

*Hargreaves S*, Rustage K*, Nellums LB*, McAlpine A, Pocock N, Devakumar D, Aldridge RW, Abubakar I, Kristensen KL, Himmels JW, Friedland JS, Zimmerman C*

**Joint first authors*

Section of Infectious Diseases and Immunity, Imperial College London; and The Institute for Infection and Immunity, St George's, University of London, London, UK (Hargreaves S FRCPE, Rustage K MSc, Nellums LB PhD; JW Himmels MD, Friedland JS FMedSci); Department of Global Health and Development, London School of Hygiene & Tropical Medicine (McAlpine A PhD); United Nations University, Kuala Lumpur, Malaysia (Pocock N PhD); UCL Institute for Global Health, London (Devakumar D PhD, Abubakar I FRCP); UCL Institute for Health Informatics, London (Aldridge R PhD); Danish Research Centre for Migration, Ethnicity and Health, University of Copenhagen (Kristensen KL); Gender, Violence and Health Centre, London School of Hygiene & Tropical Medicine, London (Zimmerman C PhD).

*Correspondence to: Dr Sally Hargreaves*

*Section of Infectious Diseases and Immunity, Imperial College London; and The Institute for Infection and Immunity, St George's, University of London, London, UK*

*s.hargreaves@sgul.ac.uk; s.hargreaves@imperial.ac.uk*

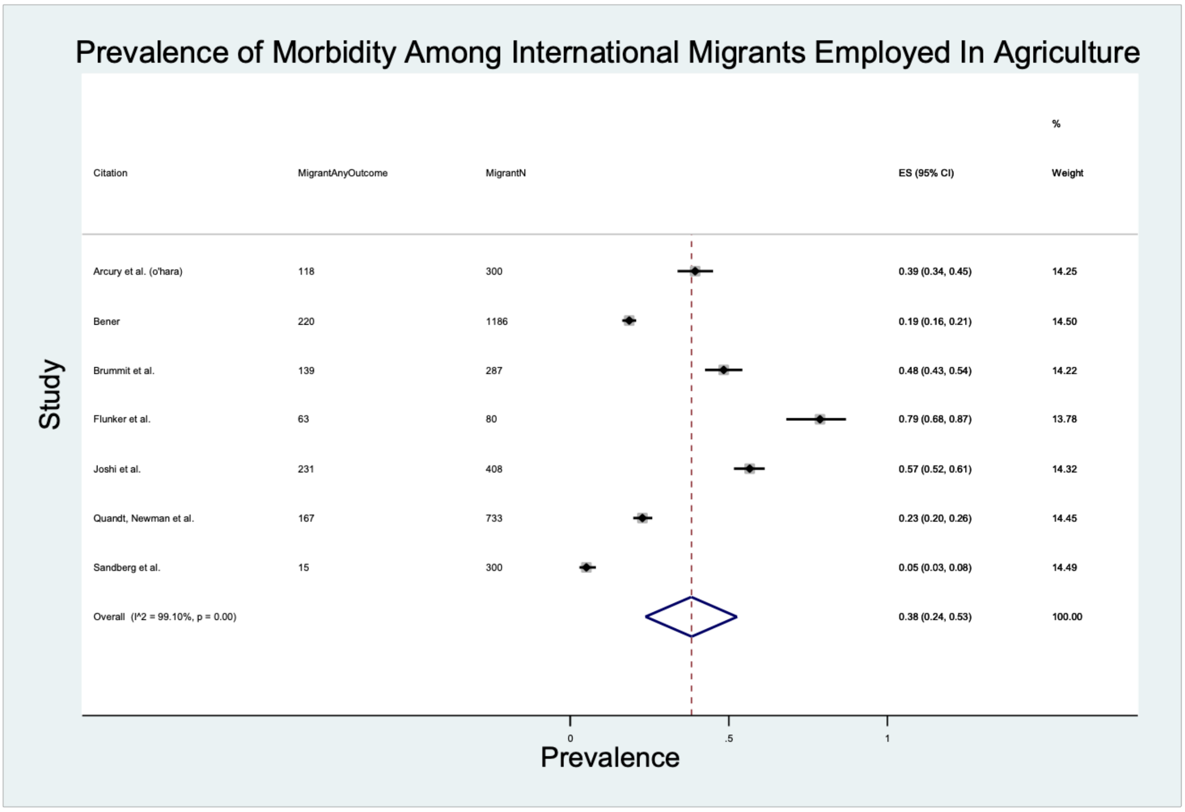


Supplementary Figure 1. Forest plot: Prevalence of morbidity among international migrant workers employed in agriculture

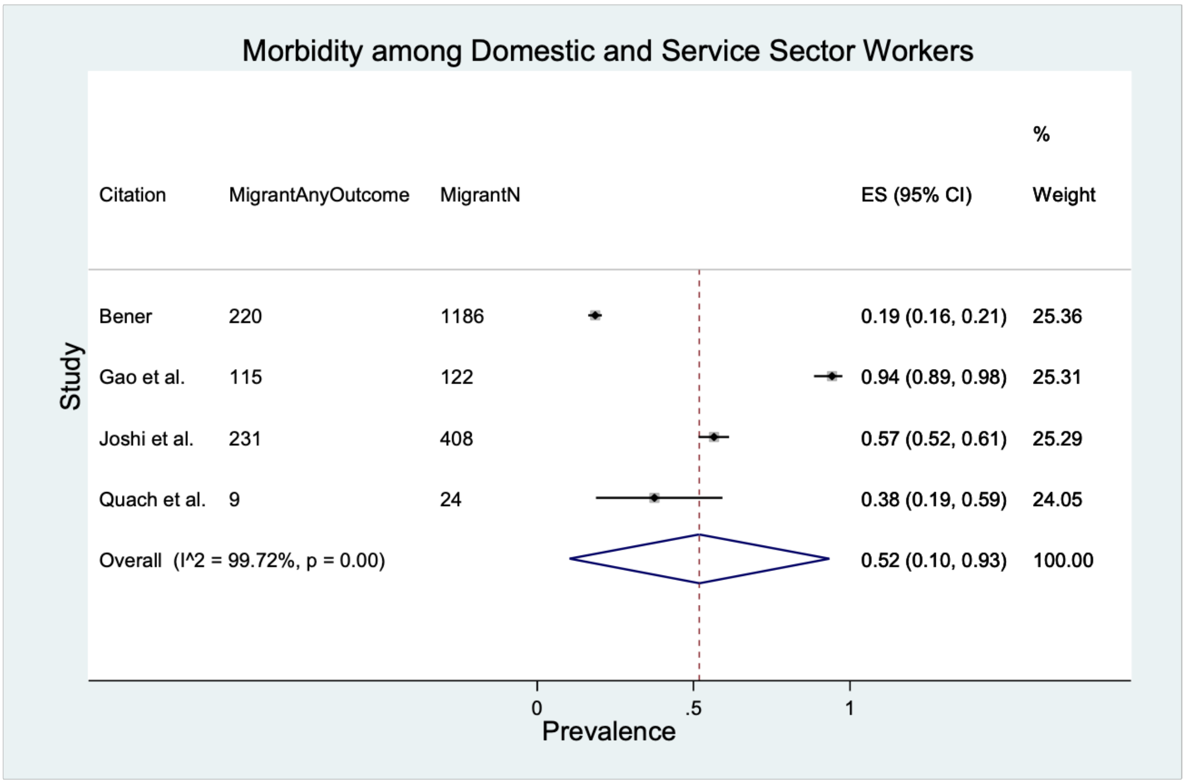


Supplementary Figure 2. Forest plot: Prevalence of morbidity among international migrant domestic and service sector workers

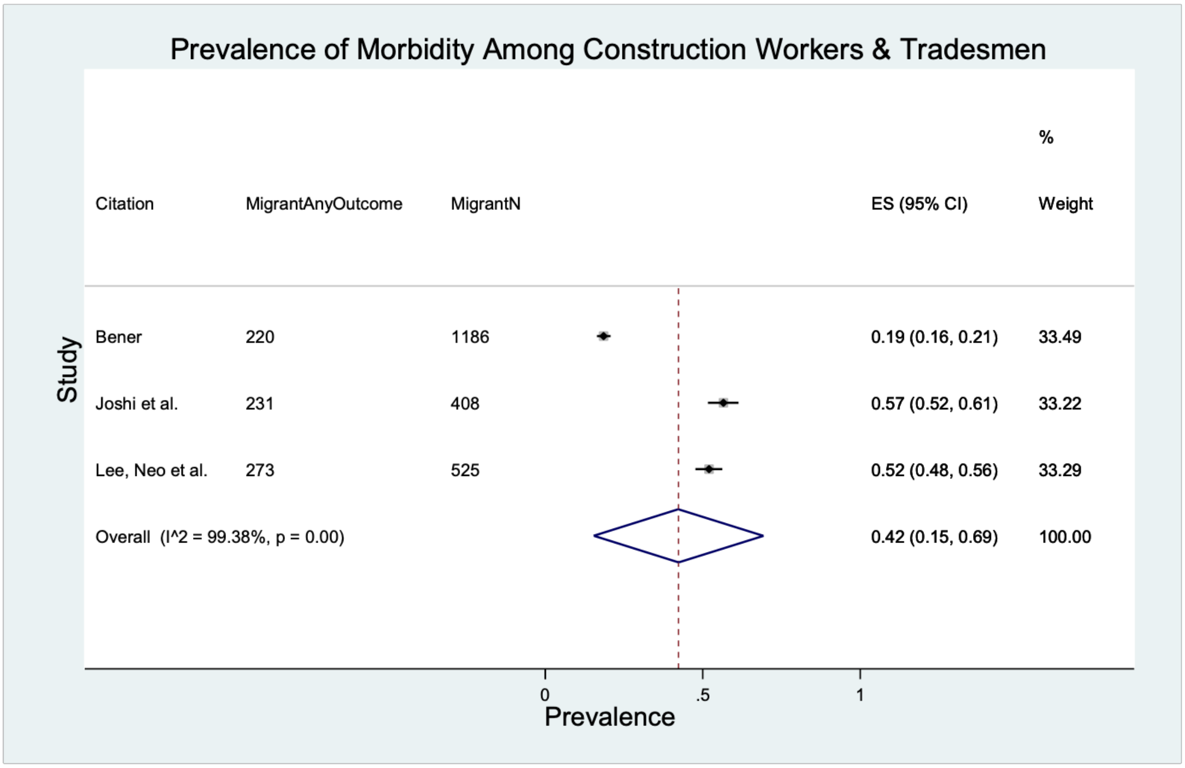


Supplementary Figure 3. Forest plot: Prevalence of morbidity among international migrant trade and construction workers

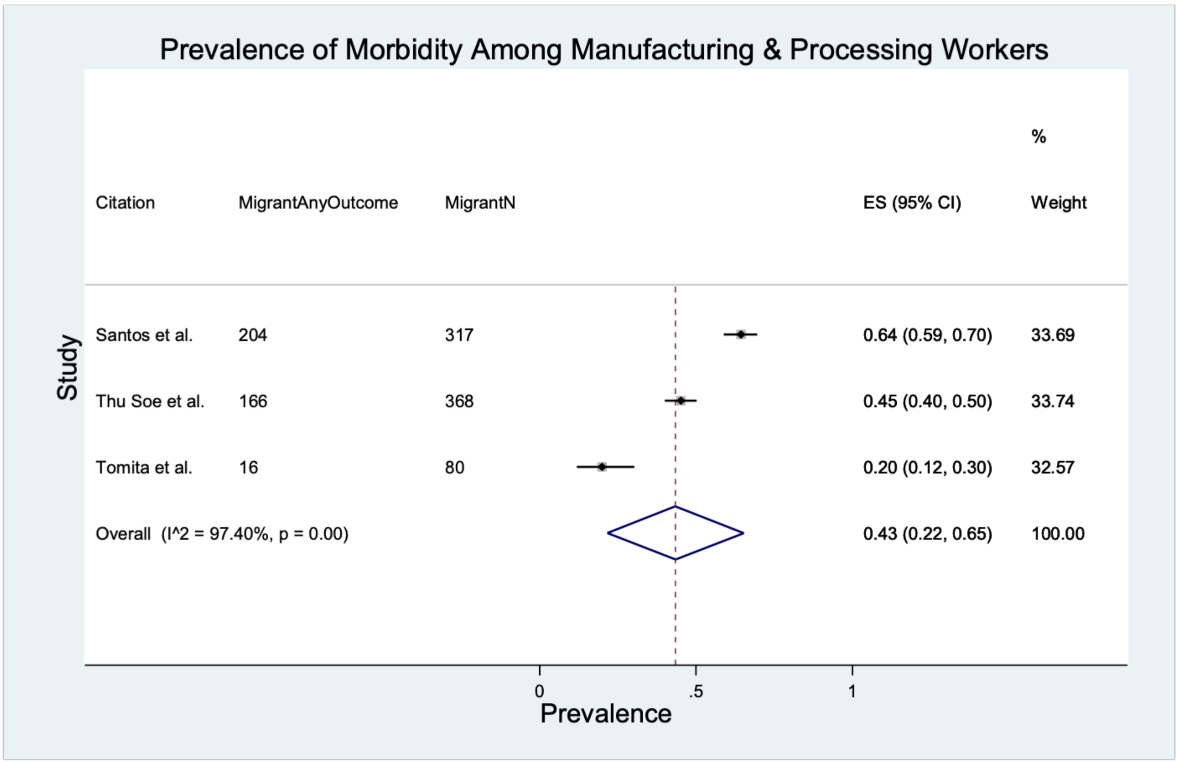


Supplementary Figure 4. Forest plot: Prevalence of morbidity among international migrant manufacturing and processing workers

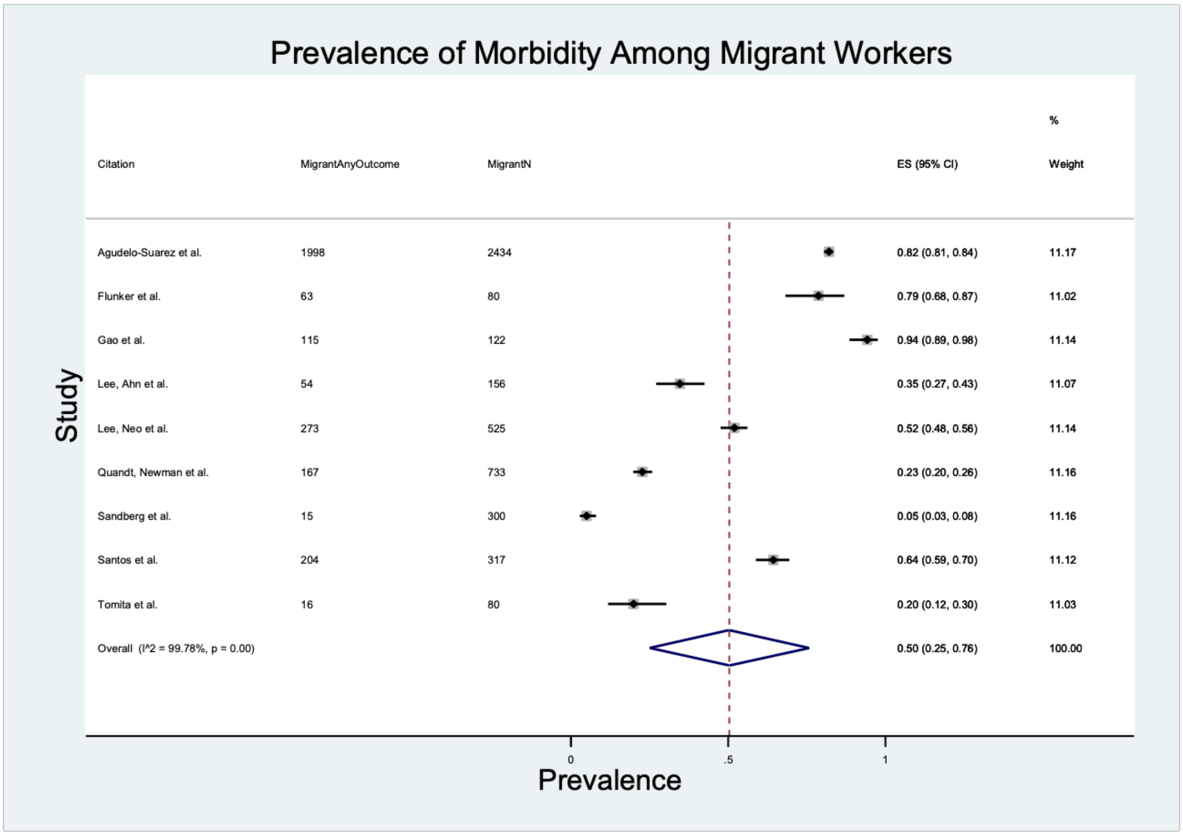


Supplementary Figure 5. Forest plot: Sensitivity analysis of prevalence of morbidity among international migrant workers

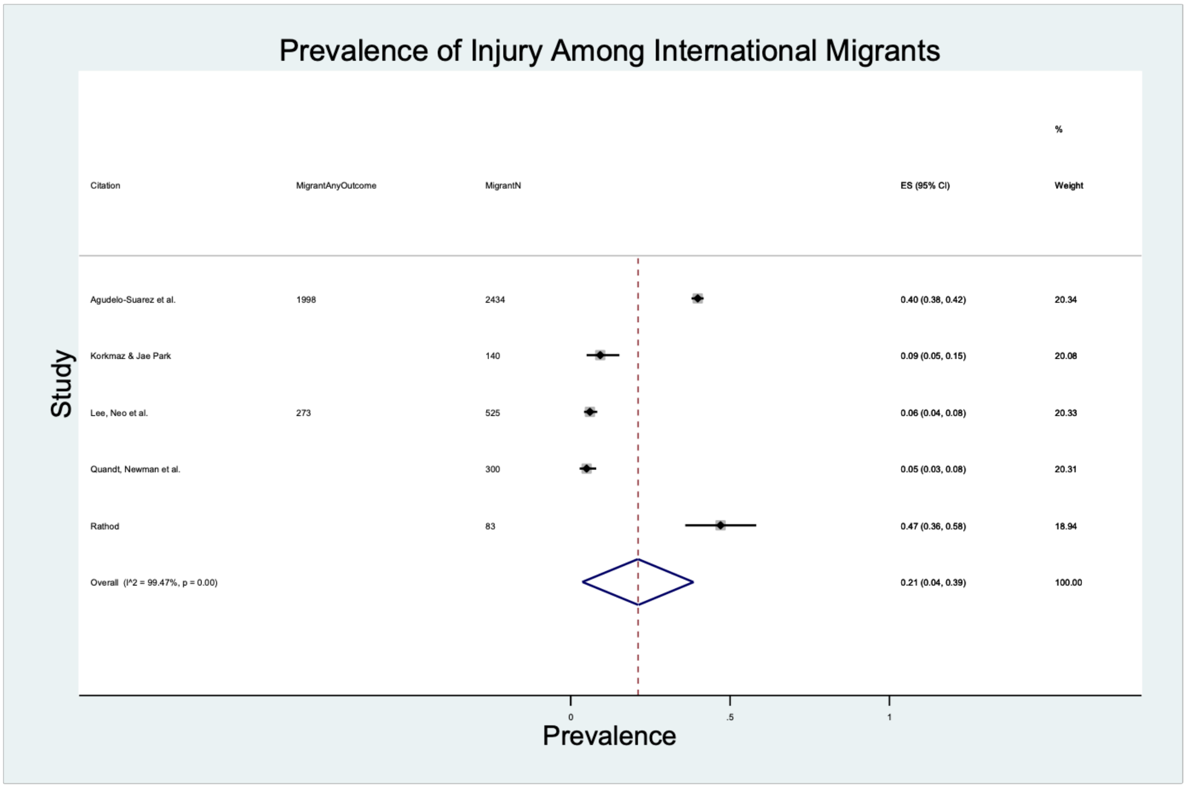


Supplementary Figure 6. Forest plot: Sensitivity analysis of prevalence of injury among international migrant workers

**Appendix 2. Search Strategy**

***keywords only, not MESH**

1. Labo?r migra* OR migrant work* OR (foreign* OR non-native OR transient OR migrant)adj2(work*) OR foreign-work* OR immigrant work* OR economic migra* OR economic immigra* OR traffick*

AND

2. (Occupation* OR work* OR employ* OR industr* OR minin* OR quarrying OR forestr* OR rubber plantation* OR plantation* OR palm oil OR fisher* OR fishing OR manufacturing OR retail* OR construction OR agricultur* OR farm* OR brick kiln* OR domestic work*)adj3(health OR injur* OR disease* OR hazard* OR exposure* OR accident* OR hygiene OR safety OR medicine OR trauma* OR fatalit* OR death* OR ill* OR syndrom* OR wound* OR fatigue OR risk* OR exposure OR safety OR disabilit* OR morbidit* or mortalit* OR homicid* OR infect* OR disorder* OR pain* OR ache*)

OR

3. (Occupation* OR work* OR employ* OR industr* OR minin* OR quarrying OR forestr* OR rubber plantation* OR plantation* OR palm oil OR fisher* OR fishing OR manufacturing OR retail* OR construction OR agricultur* OR farm* OR brick kiln* OR domestic work*)adj3(respirat* OR musculoskeletal OR cardiovascular OR cancer* OR hypertensi* OR chronic OR obstructive OR hearing loss* OR dermat* OR allerg* OR repetitive injur* OR silicosis OR pneumoconiosis OR puncture* OR laceration OR electrocution* OR fall* OR drowning OR pneumonia OR exhaustion OR broken bone* OR toxic* OR poison* OR puncture OR burn* OR parasite* OR tendon* OR pesticide* OR insecticide OR dehydration OR ocular OR eye* OR asthma OR bronchitis OR pulmonary)

OR

4. (Occupation* OR work* OR employ* OR industr* OR minin* OR quarrying OR forestr* OR rubber plantation* OR plantation* OR palm oil OR fisher* OR fishing OR manufacturing OR retail* OR construction OR agricultur* OR farm* OR brick kiln* OR domestic work*)adj3(mental* OR psychosocial OR neurotic OR mood)adj3(problem* OR disorder* OR ill* OR health OR stress* OR wellbeing OR well-being)

5. (Occupation* OR work* OR employ* OR industr* OR minin* OR quarrying OR forestr* OR rubber plantation* OR plantation* OR palm oil OR fisher* OR fishing OR manufacturing OR retail* OR construction OR agricultur* OR farm* OR brick kiln* OR domestic work*)adj3(anxiety OR depress* OR psychiatric OR bipolar OR psychos* OR schizophren* OR fear OR guilt OR hostil* OR shame OR suicid*)

NOT

5. Sexual health OR sexually transmitted disease* OR sexually transmitted infection* OR molecul* OR membrane* OR cell* OR protein* OR sex-traffick* OR sex traffick* OR sex work*
